# The high efficacy of claudin18.2-targeted CAR-T cell therapy in advanced pancreatic cancer with an antibody-dependent safety strategy

**DOI:** 10.1016/j.ymthe.2025.01.012

**Published:** 2025-01-10

**Authors:** Guocheng Zhong, Xiaomin Zhang, Ruocong Zhao, Zheng Guo, Chenguang Wang, Chuan Yu, Dongzhe Liu, Ke Hu, Yujie Gao, Bochen Zhao, Xianhao Liu, Xuanren Shi, Lei Chen, Yisheng Li, Li Yu

**Affiliations:** 1 Department of Hematology and Oncology, Shenzhen University General Hospital, International Cancer Center, Shenzhen Key Laboratory, Hematology Institution of Shenzhen University, Shenzhen University Health Science Center, Shenzhen University, Shenzhen, China; 2 Guangdong Key Laboratory for Biomedical Measurements and Ultrasound Imaging, National-Regional Key Technology Engineering Laboratory for Medical Ultrasound, School of Biomedical Engineering, Shenzhen University Medical School, Shenzhen, China; 3 Shenzhen Haoshi Biotechnology Company, Shenzhen, China; 4 Shenzhen University-Haoshi Cell Therapy Institute, Shenzhen, China

**Keywords:** CAR-T cell therapy, advanced pancreatic cancer, claudin18.2, EGFR, cetuximab

## Abstract

Pancreatic cancer (PC) is one of the most lethal digestive system tumors. Claudin18.2 is highly expressed in PC tissue and could serve as a suitable target for CAR-T therapy. In the present study, we reported the utilization of tEGFR-expressing claudin18.2-targeted CAR-T cells to treat 3 patients with advanced PC. Intriguingly, all 3 patients achieved disease remission after CAR-T cell infusion, with 1 complete remission (CR) and 2 partial remissions (PRs). However, gastric mucosal injury was observed, which was recognized as on-target off-tumor toxicity (OTOT) and may be due to the expression of claudin18.2 on normal gastric tissues. To control the severe OTOT in patient 3, cyclophosphamide and cetuximab were administered to deplete CAR-T cells, and they successfully controlled OTOT. Single-cell transcriptome and T cell receptor sequencing revealed the objective alterations of CAR-T cell clones after cetuximab treatment. Collectively, the present study showed the robust anti-tumor activity of claudin18.2-targeted CAR-T cells against PC and reported the feasibility of the antibody-dependent safety switch strategy to control the OTOT caused by CAR-T cells in patients. Our study may pave the way for the development of a novel strategy to treat patients with advanced PC in the future.

## Graphical abstract

**Figure:**
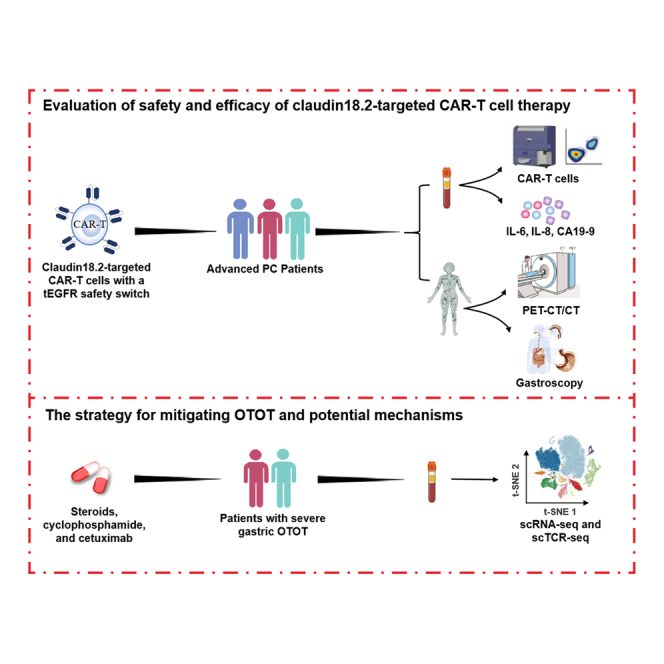

## Teaser

Zhong and colleagues report that claudin18.2-targeted CAR-T cell therapy showed satisfactory clinical efficacy in patients with advanced pancreatic cancer. Moreover, incorporating the antibody-dependent safety switch into CAR-T cells is an effective strategy for mitigating serious on-target off-tumor toxicity induced by CAR-T cell therapy, which may augment the safety profile of CAR-T cell therapy.

## Introduction

In recent years, the application of chimeric antigen receptor T (CAR-T) cells has revolutionized the clinical treatment of hematological malignancies, including B cell acute lymphoblastic leukemia and B cell non-Hodgkin lymphoma.^1^^,^^2^^,^^3^ However, the efficacy of CAR-T cells in solid cancers remains uncertain and generally unsatisfactory.^4^ CARs are recombinant molecules typically composed of an extracellular single-chain variable fragment (scFv) that recognizes tumor-associated antigens (TAAs), a transmembrane domain, and T cell-activating intracellular domains. The effectiveness of CAR-T cell therapy relies on the recognition of TAAs on the surface of cancer cells. Multiple TAAs in solid cancers have been reported, and the anti-tumor efficacy of CAR-T cells targeting these antigens has been evaluated in preclinical or clinical stages.^5^^,^^6^ It is widely accepted that the expression profile and tissue specificity of TAAs have a profound impact on both the efficacy and adverse effects of CAR-T therapy.

Pancreatic ductal adenocarcinoma (PDAC), commonly referred to as pancreatic cancer (PC), is one of the most aggressive solid malignancies, characterized by a high mortality rate, poor prognosis, and a strong tendency to metastasize. The median survival time of PC patients is less than 1 year, and the 5-year survival rate is below 10%.^7^^,^^8^ Therefore, there is an urgent need to explore new therapeutic approaches for PC. Current research on new targets for CAR-T cell therapy in PC mainly focuses on mesothelin (MSLN), prostate stem cell antigen, CD133, CD276, human epidermal growth factor receptor 2, epidermal growth factor receptor (EGFR) mutant type III, mucin 1, and claudin18.2.^9^^,^^10^^,^^11^ Of note, claudin18.2, an isoform of claudin18 and a member of the tight junction protein family, is often highly expressed in various gastrointestinal tumors, including gastric cancer (GC)/gastroesophageal junction cancer, colon cancer, biliary tract cancer, and PC.^12^ In particular, claudin18.2 is highly expressed in 65%–70% of PC patients, making it an attractive target for immunotherapy, including monoclonal antibodies and CAR-T cell therapy.^13^^,^^14^ CT041, a second-generation claudin18.2-specific CAR-T therapy, has showed promising efficacy with acceptable toxicity in phase 1 trials.^15^^,^^16^ Hence, claudin18.2-specific CAR-T therapy is a potential therapeutic strategy for PC, although its efficacy and toxicity require further verification in large-scale clinical studies.

CAR-T cell therapy is often accompanied by adverse effects, such as cytokine release syndrome^17^ and immune effector cell-associated neurotoxicity syndrome in the treatment of hematological malignancies.^18^ Additionally, on-target off-tumor (OTOT) toxicities may be observed when TAAs targeted by CAR-T cells are also expressed in normal tissues and organs. OTOT, in particular, limits the therapeutic dose of CAR-T cells and poses a significant challenge to the clinical application of CAR-T therapy in solid cancers.^19^^,^^20^ To mitigate OTOT and other toxicities associated with CAR-T cells, safety switch elements, such as inducible caspase-9^21^^,^^22^ and a truncated version of EGFR (tEGFR), have been incorporated into the CAR structure.^23^^,^^24^^,^^25^ The inducible caspase-9 safety switch depends on chemical drugs to activate suicide genes co-expressed in CAR structure, while the tEGFR safety switch relies on antibody-dependent cell-mediated cytotoxicity (ADCC) to eliminate CAR-T cells. Cetuximab, an anti-EGFR antibody approved for the treatment of EGFR^+^ colorectal cancer, can be utilized to deplete tEGFR-expressing CAR-T cells, which has already been evaluated in pre-clinical models.^24^^,^^25^ However, the feasibility of this antibody-dependent safety switch requires further validation in clinical studies.

In our study, we report the outcomes of 3 patients with advanced PC who were treated with claudin18.2-targeted CAR-T cells incorporating the tEGFR safety switch gene. All 3 patients achieved disease remission after CAR-T infusion, including 1 with complete remission (CR) and 2 with partial remission (PR). However, all 3 patients experienced gastric OTOT, as evidenced by gastric mucosal injury, which was likely due to the expression of claudin18.2 in normal gastric tissue. In particular, patient 3 suffered from the most severe gastric OTOT, which was effectively controlled using the EGFR-targeted antibody cetuximab, with no adverse reactions observed during its administration. Therefore, our study suggests that claudin18.2-targeted CAR-T cells are highly effective against PC, and antibody-dependent safety switches may be an effective strategy for eliminating CAR-T cells in cases of severe adverse events such as OTOT. The findings of this study provide a potentially effective and safe strategy for the treatment of patients with advanced PC and contribute to our understanding of OTOT induced by CAR-T cell therapy in solid tumors.

## Results

### Objective responses in 3 PC patients treated with claudin18.2-targeted CAR-T cells

**Figure 1:**
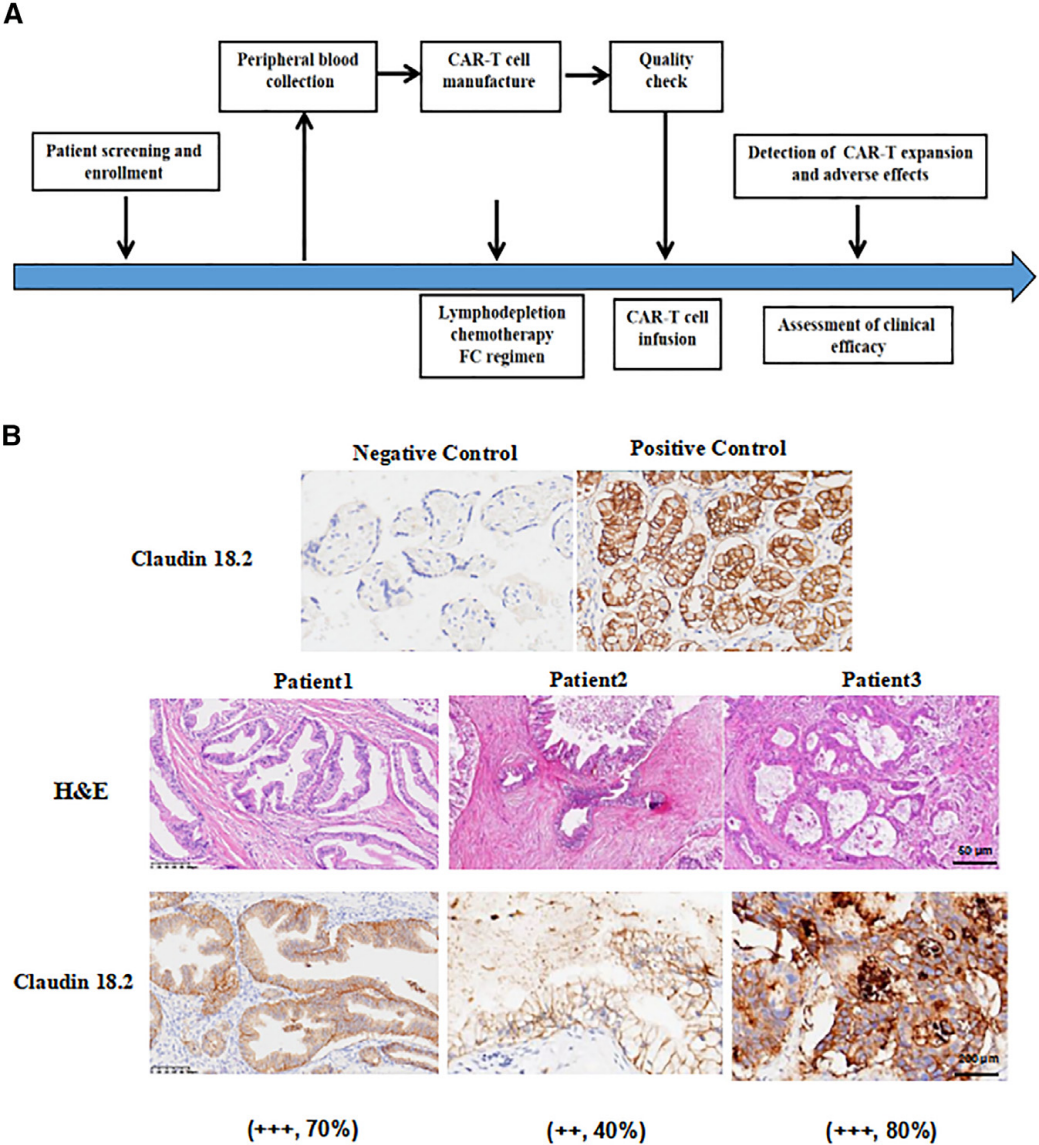
Schematic diagram of experiments and IHC result (A) Flowchart of CAR-T cell therapy. (B) Immunohistochemistry of claudin18.2 expression in tumor pathological sections/tissues of 3 patients.

**Figure 2:**
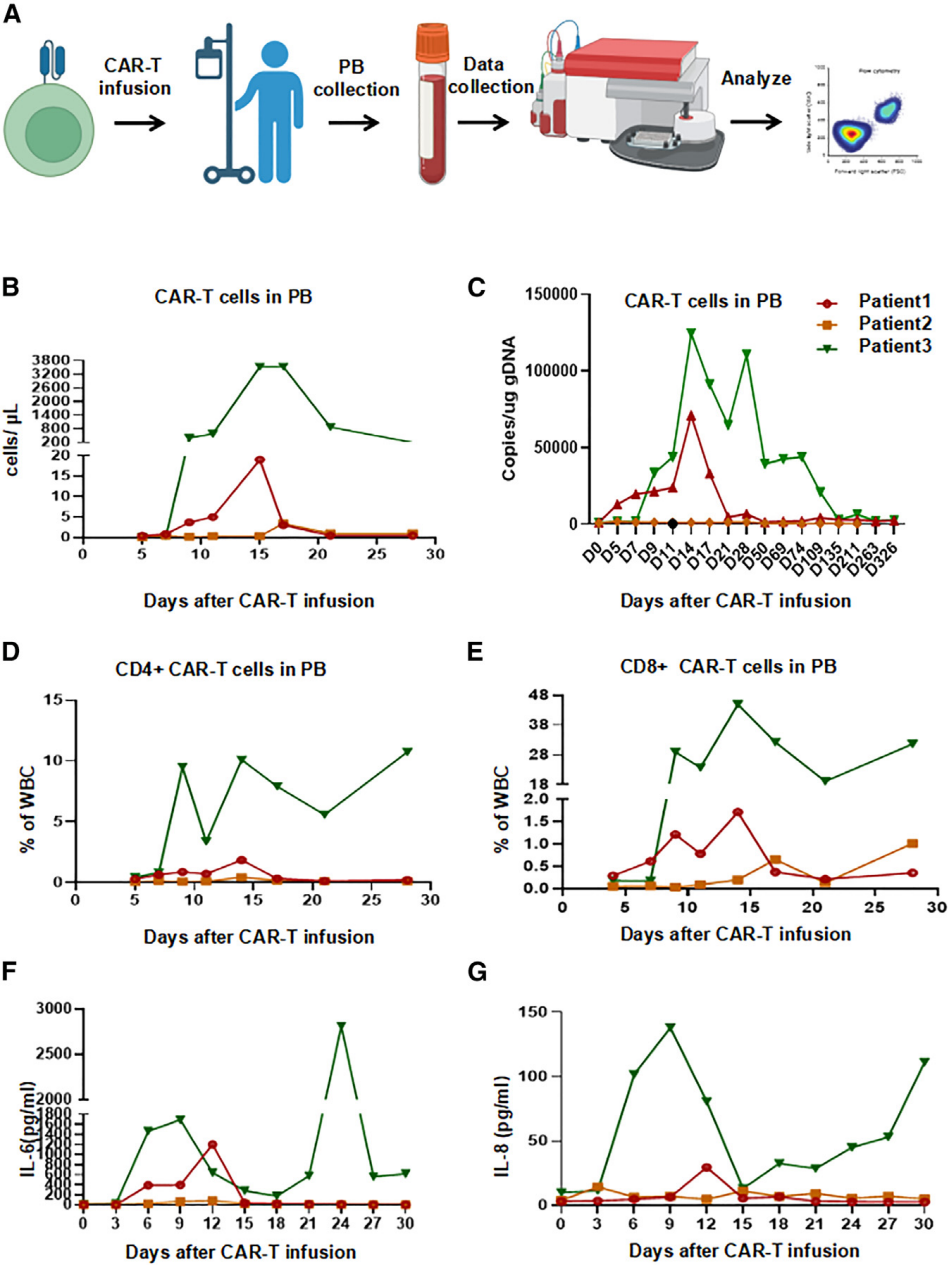
Detection of inflammatory cytokines and CAR-T cells in PB of patients (A) Schematic diagram of experiments. (B) Absolute number of CAR-T cells in the white blood cells from 3 patients detected by FACS. (C) Copy numbers of CAR in the peripheral blood (PB) from 3 patients after CAR-T cell infusion detected by qPCR. (D) The percentage of CD4^+^ CAR-T cells in white blood cells from 3 patients. (E) The percentage of CD8^+^ CAR-T cells in white blood cells from 3 patients. (F) The levels of IL-6 in 3 patients after CAR-T cell infusion. (G) The levels of IL-8 in 3 patients after CAR-T cell infusion.

**Figure 3:**
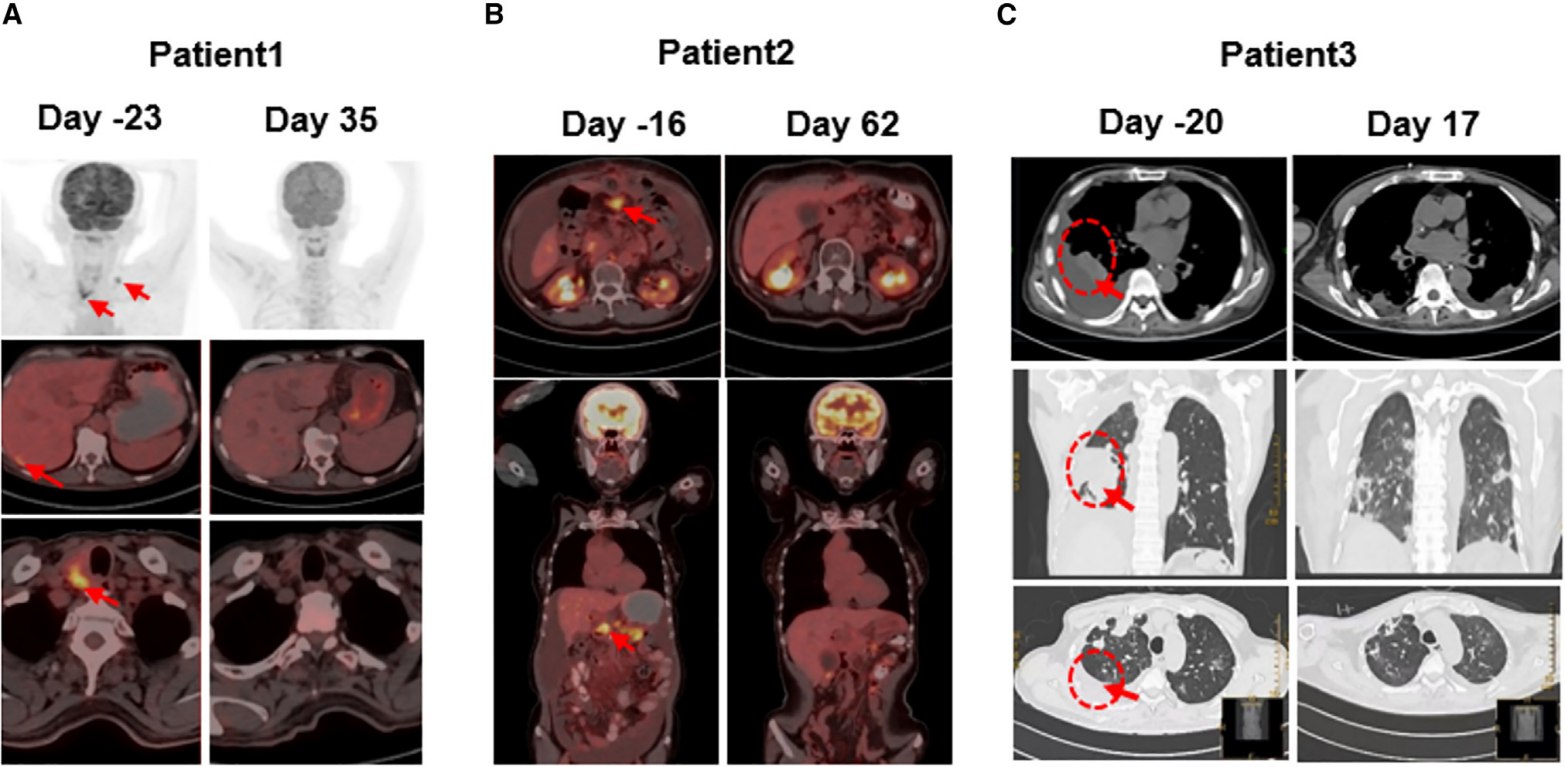
Evaluation of treatment efficacy (A) PET-CT shows that the lymph node metastases in zones III and V of the neck disappeared 35 days after CAR-T cell infusion in patient 1, as well as the right posterior lobe of the liver or intrahepatic metastatic lesions. (B) PET-CT shows that the recurrent lesion at the surgical resection site was eliminated 62 days after CAR-T cell infusion in patient 2. (C) CT scan shows the disappearance of pulmonary metastases and pleural effusion 17 days after CAR-T cell infusion in patient 3.

**Figure 4:**
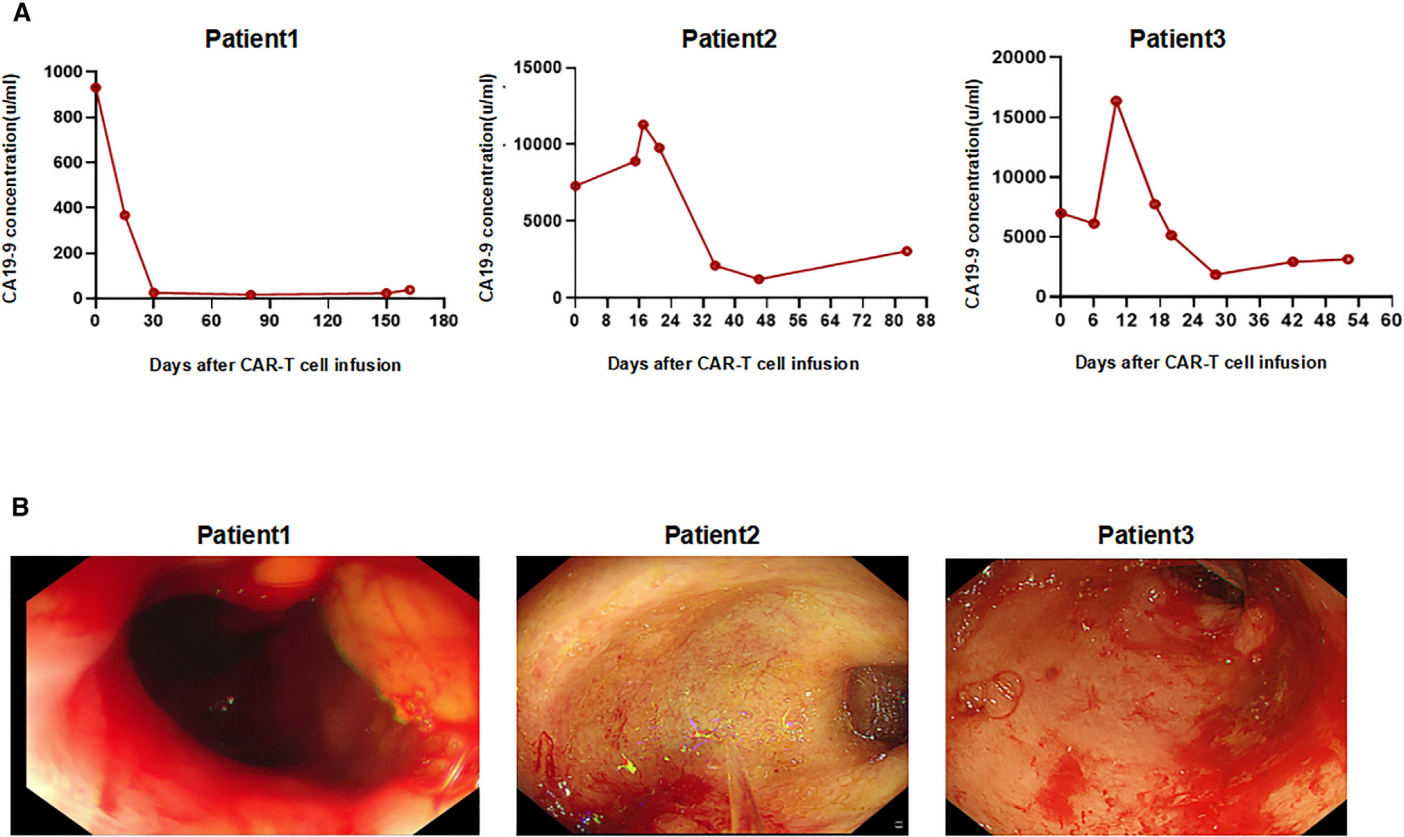
Tumor marker CA19-9 and gastric toxicity (A) The levels of tumor marker CA19-9 in the PB of all 3 patients were significantly decreased 30 days after CAR-T cell infusion. (B) Gastroscopy showed the gastric mucosal injury after claudin18.2-targeted CAR-T cell therapy, including edema, erosion, and bleeding. Among them, patient 3 suffered from the most severe gastric mucosal injury.

We conducted a pilot clinical trial study in 3 patients with advanced PDAC, and all patients received lymphodepleting chemotherapy with FC regimen (fludarabine 50 mg/m^2^ day −5 to day −3, cyclophosphamide 400 mg/m^2^ day -5 to day -3) (Figure 1A). Patient 1 was described in our previous report.^26^ He is a 72-year-old man who underwent pancreaticoduodenectomy and second-line chemotherapy, and his tumor tissue showed high levels of claudin18.2 expression (+++, positive tumor cell rate ≥70%) (Figure 1B). After lymphodepleting chemotherapy, he was treated with a single infusion of claudin 18.2-targeted CAR-T cells at a dose of 1.2 × 10^6^ cells/kg. CAR-T cell expansion reached peak levels of 19 cells/μL in peripheral blood (PB) on day 14 as detected by fluorescence-activated cell sorting (FACS) and qPCR (Figures 2B and 2C), with a balanced distribution of CD4^+^ and CD8^+^ T cells (Figures 2D and 2E), and the levels of IL-6 and IL-8 reached their peak on day 12 (Figures 2F and 2G). Surprisingly, positron-emission tomography-computed tomography (PET-CT) showed CR of tumor lesions 35 days after CAR-T cell therapy, including cervical lymph node and liver metastases (Figure 3A). The levels of the tumor marker CA19-9 were significantly decreased from 1,100 to 25.85 U/mL and reached the normal range 1 month after CAR-T cell infusion (Figure 4A). After clinical assessment, the patient achieved CR 1 month after CAR-T cell therapy (Figure 3A). Eight months after CAR-T cell therapy, the disease relapsed, and tissue biopsy and immunohistochemical (IHC) detection showed extremely low expression of claudin18.2 in this relapsed cancer tissue, suggesting the antigen escape of cancer cells from CAR-T cell immunosurveillance. Meanwhile, the relapsed tumor exhibited high expression of MSLN, which could be targeted by MSLN-specific CAR-T cells as subsequent therapy (Figure S1). However, extensive metastases were subsequently found in the liver and abdominal cavity, which compressed the intrahepatic bile ducts and resulted in liver function abnormalities, jaundice, and biliary infection. Consequently, the anti-tumor treatment was stopped, and the patient was given the best supportive care. Finally, the patient died from multi-organ failure caused by widespread recurrence and metastases of PC 14 months after CAR-T infusion.

Patient 2 is a 59-year-old female with advanced PC, and she underwent pancreatic body and tail resection with splenectomy and multi-line chemotherapy. The tumor tissue showed substantial expression of claudin18.2 (++, positive tumor cell rate ≥40%) (Figure 1B). After lymphodepleting pretreatment, she was infused with claudin18.2-targeted CAR-T cells at a dose of 1.3 × 10^6^ cells/kg. CAR-T cell expansion reached peak levels of 4 cells/μL in PB on day 13 as detected by FACS and qPCR (Figures 2B and 2C), while interleukin-6 (IL-6) and IL-8 remained at low levels (Figures 2F and 2G). PET-CT showed that the recurrent lesion at the surgical resection site was eliminated 62 days after CAR-T cell infusion, and the ascites disappeared (Figure 3B). In addition, the levels of the tumor marker CA19-9 were significantly decreased from 11,290 to 1,230 U/mL (Figure 4A). This PR status was maintained for 5 months, during which no anti-tumor treatment was performed. Subsequently, the patient experienced disease progression, but she refused to receive CAR-T cell therapy again due to worry about the risk of gastric mucosal damage. The patient died from multi-organ failure caused by extensive metastases of PC 7 months after CAR-T cell infusion.

Patient 3 is a 66-year-old man with advanced PC, and he underwent pancreatic body and tail resection with splenectomy and multi-line chemotherapy. His tumor tissues showed the high expression of claudin18.2 (+++, positive tumor cell rate ≥80%) (Figure 1B). He received lymphodepleting chemotherapy (fludarabine 50 mg/m^2^ day-5 to day -3, cyclophosphamide 400 mg/m^2^ day -5 to day -3), and infused with claudin18.2-targeted CAR-T cells at a dose of 1.588 × 10^6^ cells/kg. Nine days after CAR-T cell infusion, CAR-T cells drastically expanded and reached peak levels of 4,280 cells/μL in PB on day 14 as detected by FACS and qPCR (Figures 2B and 2C), with a dominant expansion of CD8^+^ T cells (Figures 2D and 2E). CT scan showed the disappearance of pulmonary metastases and pleural effusion 17 days after CAR-T cell infusion (Figure 3C), and the levels of the tumor marker CA19-9 were significantly decreased from 16,370 to 930 U/mL (Figure 4A). However, the patient experienced severe gastric mucosal damage during CAR-T cell therapy, characterized by gastric bleeding with the excessive expansion of CAR-T cells (Figures 2B and 4B). Both conventional hemostasis and electrocoagulation hemostasis under gastroscopy were ineffective, and blood pressure progressively declined, which threatened the patient’s life. To avoid the serious OTOT, the patient was treated with 15 mg dexamethasone, 40 mg methylprednisolone, 0.2 g cyclophosphamide, and 300 mg cetuximab to eliminate the CAR-T cells. When the number of CAR-T cells in PB decreased to fewer than 10 cells/μL, gastric bleeding was relieved. However, the tumor progressed with the reappearance of pleural effusion, infection, malnutrition, thrombosis, and other complications, and the levels of CA19-9 were upregulated again. The patient died from respiratory failure caused by tumor recurrence 3 months after CAR-T cell infusion.

### Effectiveness of tEGFR-cetuximab antibody-dependent safety switch in managing OTOT caused by claudin18.2-targeted CAR-T cells

All 3 patients reported gastrointestinal adverse effects during CAR-T cell therapy. These gastrointestinal symptoms were mainly attributed to OTOT caused by claudin18.2-targeted CAR-T cells, which attacks normal gastric tissue expressing claudin18.2 (Table 1). For patients 1 and 2, gastroscopy and biopsy revealed diffuse gastric mucosal edema, hyperemia, and denudation after claudin18.2-targeted CAR-T cell therapy (Figure 4B). The symptoms were partially alleviated by proton pump inhibitors, prokinetic drugs, and gastric mucosal protectants.

**Table 1:** Patients’ information

	Patient 1	Patient 2	Patient 3
Sex	male	female	male
Age, y	71	59	66
Surgery time	June 11, 2021	November 23, 2021	August 12, 2020
Surgery type	pancreaticoduodenectomy	pancreaticocaudectomy + splenectomy	pancreaticocaudectomy + splenectomy
Claudin18.2 expression	+++, 70%	++, 40%	+++, 80%
Metastatic site	lungs, liver, abdominal, lymph nodes, cervical lymph nodes	bone, liver, abdominal cavity, and ascites	lungs, bones, abdominal cavity, and pleural effusion
Time for CAR-T infusion	November 21, 2022	March 6, 2023	June 30, 2023
Infused CAR-T no.	1.2 × 10^6^/kg	1.3 × 10^6^/kg	1.58 × 10^6^/kg
Best overall response	CR (PET-CT)	PR (PET-CT)	PR (CT)
Peak of CAR-T in PB	19 cells/μL	4 cells/μL	4280 cells/μL
Time to peak, day	14	13	14
Reduction of CA19-9	from 1,100 to 15	from 11,290 to 1,230	from 16,370 to 930
Lines of chemotherapy	3	4	5
Adverse effect	gastric mucosal congestion and edema	gastric mucosal congestion and edema	severe gastric mucosal bleeding
OS after CAR-T infusion, months	14	7	3

OS, overall survival.

**Figure 5:**
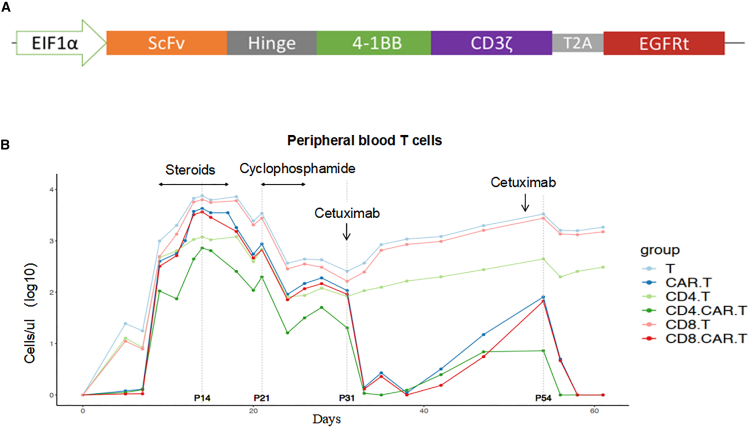
Kinetics of CAR-T cells in patient 3 (A) The structure of claudin18.2-targeted CAR with a tEGFR safety switch. (B) For patient 3, claudin18.2-targeted CAR-T cells in PB rapidly proliferated and reached a peak of 4,280 cells/μL (day 14). To inhibit the robust expansion of CAR-T cells and mitigate gastric mucosal bleeding, the patient was treated with steroids and 0.2 g cyclophosphamide twice (days 21 and 26). After the administration of cyclophosphamide, the number of T cells was rapidly reduced, including CD4^+^ T cells, CD8^+^ T cells, and CAR-T cells. In particular, the levels of CAR-T cells in the patient’s PB decreased from 870 to 90 cells/μL and from 189 to 107 cells/μL, respectively. Subsequently, due to repeated gastric mucosal bleeding, 300 mg cetuximab was administered twice (days 32 and 52) to specifically deplete the CAR-T cells, and the levels of CAR-T cells decreased from 107 to 0.4 cells/μL and from 80 to 0 cells/μL, respectively. After the last administration of cetuximab (day 52), gastric mucosal bleeding was stopped.

For patient 3, the CAR-T cells in PB began to rapidly expand, reaching 400 cells/μL (day 9, on July 9, 2023) and continuously increased, reaching a peak of 4,280 cells/μL on day 14. Severe gastric mucosal damage accompanied by gastric bleeding (grade 3 gastrointestinal reaction) occurred when CAR-T cells drastically expanded (Figure 4B). The bleeding could not be stopped with conventional and electrocoagulation hemostasis. To suppress the robust CAR-T cell expansion and control OTOT, the patient received 15 mg dexamethasone (days 7 and 8) and 40 mg methylprednisolone (days 8–16). However, the levels of CAR-T cells in PB were not significantly decreased. Therefore, patient 3 was then treated with 0.2 g cyclophosphamide twice (days 21 and 26). After cyclophosphamide intervention, the number of CAR-T cells in PB decreased from 870 to 90 cells/μL and from 189 to 107 cells/μL, respectively. Because CAR used in the present study contained a tEGFR label (Figure 5A), subsequently, 300 mg cetuximab was given twice (days 32 and 52), and the levels of CAR-T cells decreased from 107 to 0.4 cells/μL and from 80 to 0 cells/μL, respectively. Then, the gastric mucosal damage was quickly relieved. After first administration with cetuximab on August 1, 2023 (day 32), the number of CAR-T cells rapidly decreased but rebounded in 1–2 days, causing persistent gastric bleeding. After the second administration of cetuximab on August 21, 2023 (day 52), the number of CAR-T cells in PB dropped to 0 cells/μL and did not rebound (Figure 5B). Gastric bleeding was finally controlled.

These results demonstrated that the tEGFR-cetuximab antibody-dependent safety switch strategy could effectively deplete CAR-T cells when the robust expansion of CAR-T cells occurs and eventually mitigate gastric OTOT induced by claudin18.2 targeted CAR-T cell therapy.

### Single-cell RNA and TCR sequencing analysis following CAR-T cell infusion in patient 3

**Figure 6:**
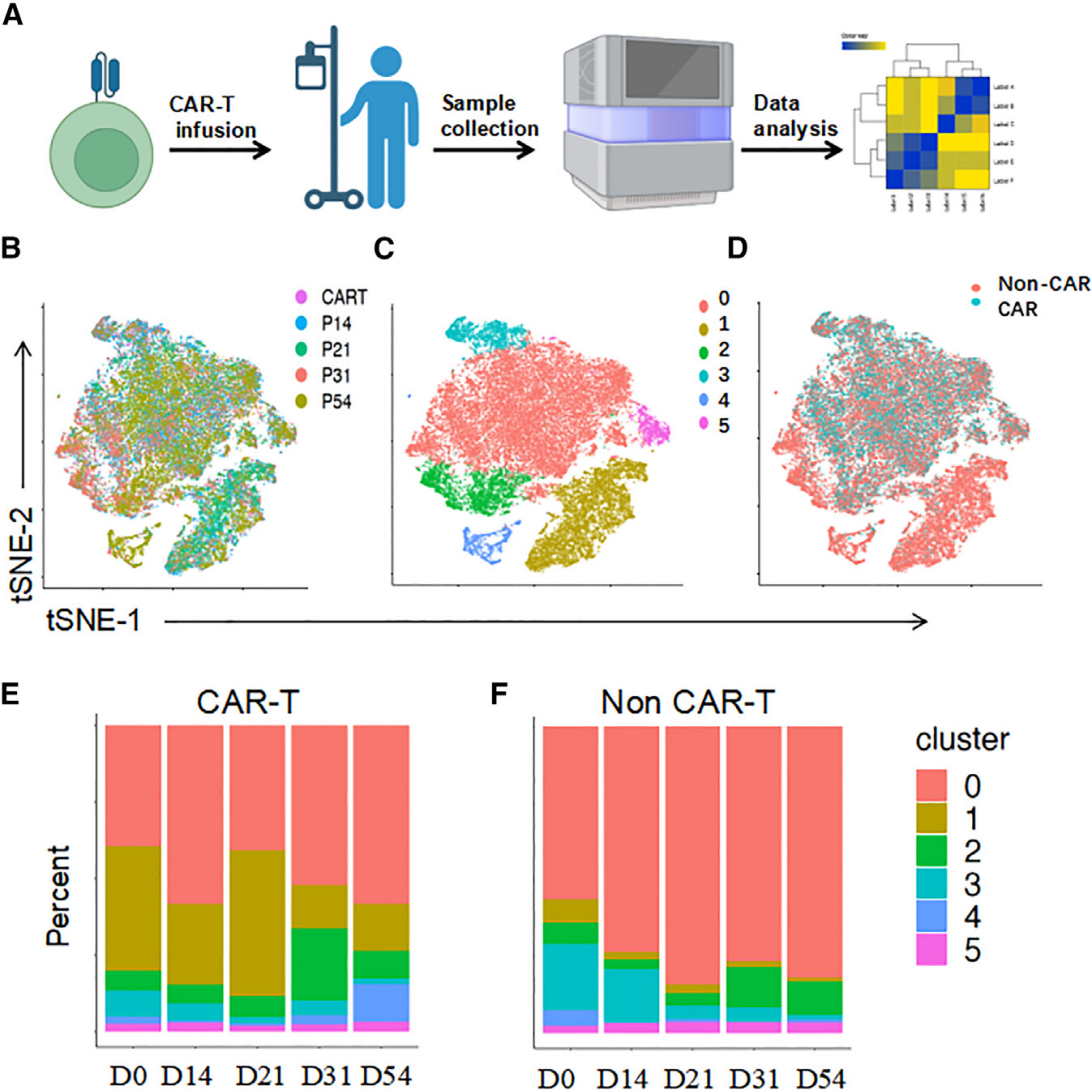
scRNA-seq analysis of CAR-T cells in patient 3 (A) Schematic representation of the experimental workflow. CAR-T cells were infused into patient 3, followed by sample collection at multiple time points (days 0, 14, 21, 31, and 54). (B) tSNE plot showing the clustering of CAR-T cells at different time points post-infusion. Six distinct clusters (0–5) were identified based on transcriptional profiles. (C) tSNE plot depicting the distribution of CAR-T cells from different time points (days 0, 14, 21, 31, and 54) across the identified clusters. Each color represents a different time point. (D) tSNE plot differentiating between CAR-T cells (teal) and non-CAR-T cells (red) within the same dataset. (E) Stacked bar plot representing the percentage distribution of CAR-T cells within each cluster (0–5) at different time points (days 0, 14, 21, 31, and 54). (F) Stacked bar plot showing the percentage distribution of non-CAR-T cells across the same clusters (0–5) at the same time points (days 0, 14, 21, 31, and 54).

To further characterize CAR-T cells at the single-cell level in patient 3, whose PB CAR-T cell count was enormous and immune response was particularly strong, we performed single-cell RNA sequencing (scRNA-seq) and T cell receptor (TCR) sequencing on CAR-T cells collected at various time points post-infusion (Figure 6A). After applying stringent filtering criteria as described in the materials and methods, we retained 2,394, 9,272, 7,991, 7,545, and 9,148 cells from day 0, 14, 21, 31, and 54 samples, respectively, with 847, 3,489, 1,835, 2,795, and 1,393 cells identified as CAR-T cells, respectively. t-Distributed stochastic neighbor embedding (tSNE) was employed to visualize the scRNA-seq data, revealing distinct distributions of PB T cells across 5 clusters at each time point (Figures 6B and 6C), with clear differentiation between CAR-T and non-CAR-T cells (Figure 6D).

Within the clusters, clusters 0, 2, 3, and 5 exhibited high expression of markers such as CCL5, GZMA, CST7, and CD8A, indicative of cytotoxic CD8^+^ T cells with robust cytotoxic functions (Figures S2A and S4). Cluster 1 was enriched for markers including STAT4, BACH2, FYN, and FOXO1, suggesting a predominance of Th1 helper T cells involved in cell-mediated immune responses and characterized by Th1-type cytokine production and signaling pathways (Figure S3A). Cluster 4 displayed elevated expression of CXCL2, CXCL8, NFKBIA, FOS, and CD3D, likely representing a subset of activated T cells comprising both CD4^+^ and CD8^+^ subsets, which are implicated in inflammatory responses and possibly early activation or effector functions (Figure S3B). The distribution of clusters 0–5 across CAR-T and non-CAR-T cell populations revealed that cluster 0 was predominant in all samples. However, the proportions of other clusters varied: cluster 1 was more prevalent in non-CAR-T cells compared to CAR-T cells, while cluster 2 was more abundant in CAR-T cells than in non-CAR-T cells (Figures 6E and 6F).

### Clonal evolution and differential gene expression reveal CAR-T cell dynamics and stress response under sequential treatments

**Figure 7:**
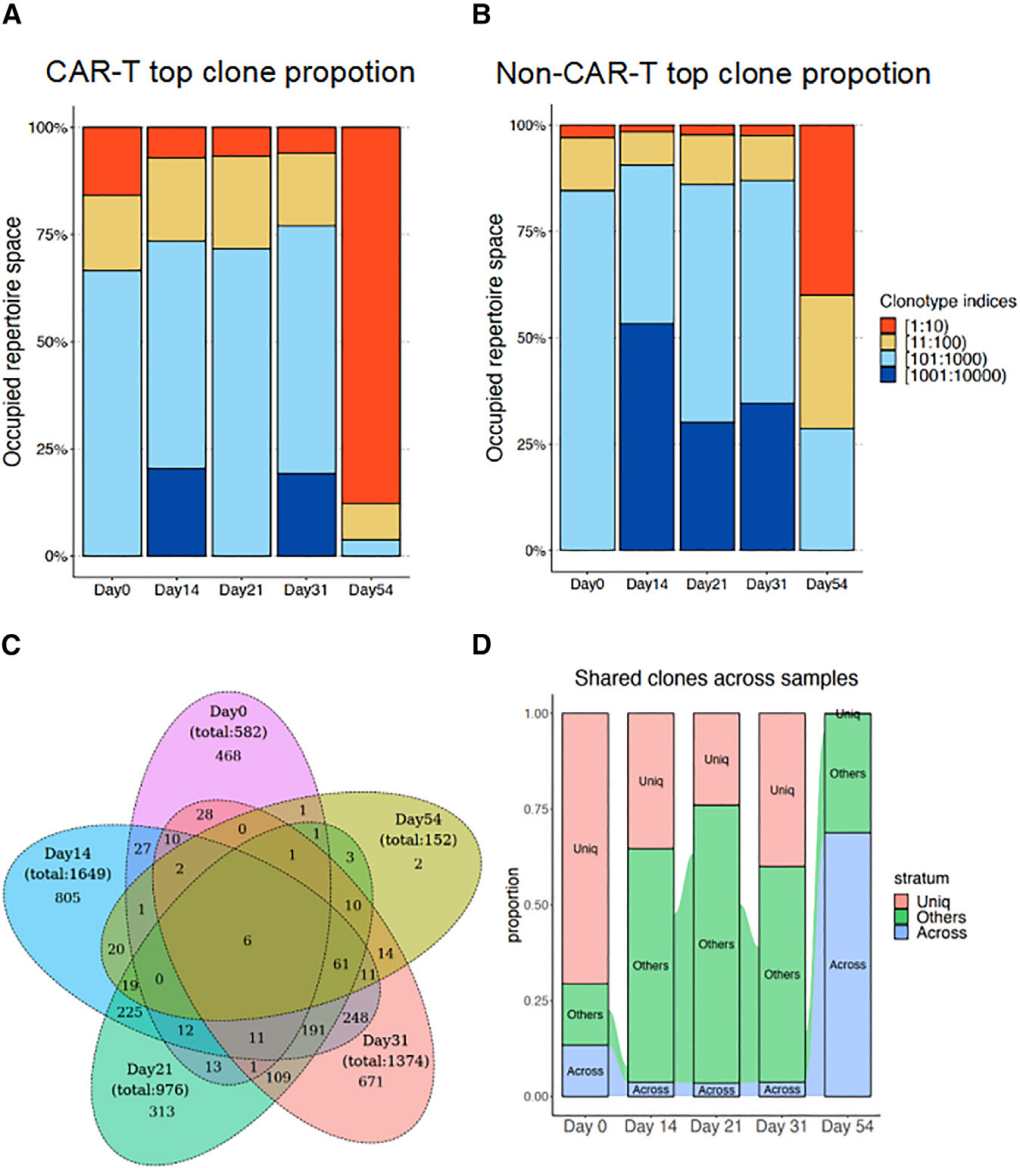
Cetuximab-induced objective alterations of CAR-T cell clones revealed by TCR sequencing (A) Bar plots depicting the top clonal proportions of CAR-T cells (left) and non-CAR-T cells (right) across different time points (days 0, 14, 21, 31, and 54). Each color represents a distinct clonotype categorized by frequency: red for the top 10 clonotypes, orange for ranks 11–100, light blue for ranks 101–1,000, and dark blue for ranks 1,001–10,000. (B) Heatmap displaying the pairwise Morisita overlap index, which quantifies the similarity in clonal distribution between different time points. The upper triangle shows the index among CAR-T cells, while the lower triangle shows the index among non-CAR-T cells. (C) Venn diagram illustrating the number of shared and unique CAR-T cell clonotypes across different time points, with overlaps representing shared clonotypes. (D) Alluvial plot showing the flow of clonotypes shared between adjacent samples over time (days 0, 14, 21, 31, and 54). Bars represent different stratum categories: “Uniq” (red) for clonotypes unique to each sample, “Across” (blue) for clonotypes consistently found across all 5 samples, and “Others” (green) for clonotypes shared between some but not all samples. The flows between bars indicate the movement and persistence of clonotypes across time points.

To further investigate clonal evolution over time, TCR repertoire analysis was performed. Non-dominant clones were prevalent in samples collected before day 31, as the CAR-T cell population underwent significant expansion without obvious clonal selection during the early stages, even under steroid or cyclophosphamide treatment. By day 54, following 2 rounds of cetuximab treatment, specific CAR-T TCR clones became highly dominant, suggesting selective expansion of these clones in response to treatment, while the majority of other clones was depleted. In contrast, non-CAR-T cell samples maintained a relatively diverse TCR repertoire over time (Figures 7A and 7B). Since the continuous generation of new T cells with distinct TCRs contributes to repertoire diversity, pairwise Morisita’s overlap index analysis was employed to assess the baseline variability in TCR repertoires. Higher overlap indices among CAR-T cell samples indicated greater consistency and clonal stability within the CAR-T TCR repertoire, whereas non-CAR-T cells exhibited much lower overlap indices across different time points, reflecting a more dynamic and diverse TCR repertoire (Figure S5A).

To more precisely track clonal dynamics over time, a Venn diagram of TCR clones across all CAR-T cell samples was generated, identifying 6 clones that were shared across all time points (Figure 7C). The proportions of CAR-T clones classified as “unique” (present in only 1 sample), “across”" (the 6 clones found in all 5 samples), and “others” were visualized using an alluvial plot (Figure 7D). The across clones represented 13.4%, 3.7%, 3.5%, 3.7%, and 68.9% of the total CAR-T cells in the day 0, 14, 21, 31, and 54 samples, respectively. The flow between adjacent samples reflects the continuity of specific clones derived from earlier samples. Notably, between days 31 and 54, only a small proportion of the day 31 clonal population persisted in the day 54 CAR-T cells, further illustrating a marked contraction in the overall CAR-T TCR repertoire. Finally, we further analyzed the distribution of differentially expressed genes (DEGs) within the preexisting TCR clones of CAR-T cells in the across group at different time points. Compared to day 14, CAR-T cells at day 0 exhibited a high expression of genes such as CCNB1 and TEX14, which are associated with pathways involved in chromosome segregation and mitotic division, indicating enhanced proliferation and cell-cycle regulation at this early stage. By day 14, CAR-T cells showed elevated expression of CXCR4 and IL7R, suggestive of strong cytokine binding and immune receptor activity. The increased expression of NR4A2, NR4A3, and TGFB1 indicated a phenotype associated with T cell exhaustion and regulatory functions, corresponding to a critical turning point in CAR-T cell treatment, marked by the peak level of CAR-T cells in the blood and a shift toward a more regulated immune response (Figures S6A–S6C). A significant number of DEGs were detected between days 31 and 54 following the switch to cetuximab treatment. Compared to day 54, genes such as HSPA1A, HSPA1B, and HSP90AA1 were highly expressed on day 31, with pathway enrichment in heat shock response, transcriptional regulation, and apoptotic regulation. This suggests that selected T cell functions were actively engaged in stress responses under cyclophosphamide treatment. In contrast, no genes were found to be upregulated on day 54. Notably, the exogenous gene pCDHEXO (the lentiviral expression cassette encoding CAR and tEGFR) was significantly downregulated compared to day 31, which might indicate that cetuximab is not effective on EGFR-low CAR-T cells (Figures S7A and S7B).

## Discussion

In recent years, CAR-T cell therapy has emerged as a highly effective method of anti-tumor treatment, and it has achieved unprecedented efficacy in relapsed/refractory hematological malignancies. However, CAR-T cell therapy in solid tumors has proven to be challenging.^4^ The lack of specific target antigens and the immunosuppressive tumor microenvironment, as well as side effects, are non-negligible issues when CAR-T cell therapy is applied in solid tumors.^6^ Most of the tumor antigens in solid tumors are tumor-associated antigens, which are also expressed on normal organs and tissues. Therefore, CAR-T cells could also attack normal tissues, and OTOT induced by CAR-T cell therapy is more likely to occur in solid tumors.^23^

PC is a highly malignant tumor in the digestive system with a poor prognosis, and conventional therapeutic approaches for PC show their relatively limited efficacy. Encouragingly, claudin18.2-targeted CAR-T cell therapy has been confirmed to be an effective strategy for the treatment of advanced PC. At present, CARsgen Therapeutics Co., Ltd. has performed 2 exploratory clinical trials of claudin18.2-targeted CAR-T cell therapy for the treatment of gastrointestinal cancers (these studies were registered at ClinicalTrials.gov: NCT04581473 and NCT03874897). These 2 multi-center, open-label phase 1 trials have shown the tolerable safety profile and encouraging clinical efficacy of claudin18.2-targeted CAR-T cell therapy in 32 gastrointestinal cancer and 5 refractory metastatic PC patients with an overall response rate of 48.6% and a median progression-free survival rate of 3.7 months. However, the efficacy in GC is significantly better than PC and other cancers.^15^^,^^16^ Previously, our group developed second-generation claudin18.2-targeted CAR-T cells for the treatment of advanced PC in a phase 1 clinical trial (this study was registered at ClinicalTrials.gov: NCT05620732).^27^ In this study, 3 patients with advanced PC were enrolled. Patient 1 achieved CR and sustained for 8 months, while patients 2 and 3 achieved PR. Unfortunately, all 3 patients developed gastric mucosal injury during CAR-T cell therapy, which may be due to the expression of claudin18.2 on normal gastric cells, and presented with gastrointestinal symptoms, such as poor appetite, nausea, vomiting, and abdominal pain. Compared with the previous phase 1 study, the response rate of our study was higher, and the gastric OTOT also appeared to be more severe (patients 1 and 2 suffered from grade 2 gastrointestinal adverse events, and patient 3 developed grade 3 gastrointestinal adverse events); the limitation of our study was the small number of patients. We speculate that these differences may be due to the different co-stimulatory signals utilized in these 2 studies, since our study utilized 4-1BB as the co-stimulatory signal, while the CT041 study utilized CD28 co-stimulation.^15^^,^^16^ It has been recognized that 4-1BB co-stimulatory signal causes less CAR-T cell exhaustion and triggers more persistent anti-tumor activity.^26^^,^^28^^,^^29^ Therefore, 4-1BB signal may contribute to the higher efficacy and more severe OTOT in the present study.

The claudin18.2-targeted CAR-T cells used in our study incorporate a truncated EGFR element, which serves dual purposes: it allows for the detection of CAR transduction efficiency via flow cytometry and enables the analysis of CAR-T cell kinetics in PB. Additionally, the tEGFR functions as a safety switch, allowing cetuximab to target and deplete CAR-T cells through ADCC. Although preclinical studies have suggested that cetuximab could effectively eliminate tEGFR-expressing CAR-T cells,^24^ whether it exhibits the same effect in patients needs to be further validated in clinical studies. Our study, particularly the case of patient 3, confirms that cetuximab could successfully deplete tEGFR-expressing CAR-T cells and control OTOT in a clinical setting. While patient 3 achieved PR following CAR-T infusion, the disease rapidly progressed after CAR-T cell depletion. This suggests that further research is needed to optimize the dosage of cetuximab, aiming to better balance anti-tumor activity with OTOT management and ultimately improve overall patient survival.

Based on the current research findings, we believe that claudin18.2-targeted CAR-T cell therapy has significant therapeutic potential in the treatment of advanced PC. Despite challenges related to adverse effects and immune escape which was also reported in our previous study, it remains one of the most promising treatment strategies for advanced PC. Moving forward, further modifications and optimization of CAR-T therapy strategies are essential to maximize its therapeutic efficacy.

## Materials and methods

### Study treatment

We conducted a phase 1 single-center clinical study of claudin18.2-targeted CAR-T cell therapy in advanced PC (this study was registered at ClinicalTrials.gov: NCT05620732) in Shenzhen University General Hospital. Written informed consent was obtained from all the participants. This study was approved by the Human Ethics Committees of the Shenzhen University General Hospital Shenzhen, China.

### Patient characteristics

Patient 1 was a 72-year-old man diagnosed with PC 2 years prior. He experienced tumor recurrence and multiple metastases following a pancreaticoduodenectomy and multiple lines of chemotherapy. The metastases included the liver, lungs, peritoneum, and cervical lymph nodes (Table 1). Due to the recurrence and metastasis after surgical resection and adjuvant therapies, the patient was recruited into a clinical trial for claudin18.2-targeted CAR-T cell therapy conducted at our center. Immunohistochemistry revealed a high level of claudin18.2 expression (+++, positive rate ≥70%) in the surgically removed tumor tissues (Figure 1B). Consequently, the patient was enrolled in the clinical trial of claudin18.2-targeted CAR-T cell therapy. After lymphodepleting chemotherapy with FC regimen (fludarabine at 30 mg/m^2^ and cyclophosphamide at 300 mg/m^2^) for 3 consecutive days, the patient received an infusion of claudin18.2-targeted CAR-T cells at a dose of 1.2 × 10^6^ cells/kg on November 21, 2022. Day 0 referred to the day on which CAR-T cells were infused.

Patient 2 was a 59-year-old female who was diagnosed with PC 2 years ago and underwent a distal pancreatectomy. Unfortunately, she developed metastases to the bone, liver, and abdominal cavity. Immunochemistry showed the positive rate of claudin18.2 was 40% (++) (Figure 1B). As a result, she was enrolled in the clinical trial of claudin18.2-targeted CAR-T cell therapy. After lymphodepleting chemotherapy with FC regimen, the patient was treated with CAR-T cells at a dose of 1.3 × 10^6^ cells/kg on March 6, 2023.

Patient 3 was a 66-year-old man diagnosed with PC 2 years prior. He also experienced tumor recurrence and multiple metastases after distal pancreatectomy, including the lung, bone, abdominal cavity, and lymph node metastases. Immunochemistry revealed the high expression of claudin18.2 (+++, positive rate ≥80%) in tumor tissues (Figure 1B). The patient was enrolled in the clinical trial of claudin18.2-targeted CAR-T cell therapy. After lymphodepleting chemotherapy with FC regimen, the patient was administrated with CAR-T cells at a dose of 1.59 × 10^6^ cells/kg on June 30, 2023.

### Hematoxylin and eosin staining and IHC

The surgically removed tumor tissues were collected and fixed in 4% paraformaldehyde for 48 h. Subsequently, the tissues were embedded in paraffin, sectioned into 4-μm-thick slices, and stained with hematoxylin and eosin (H&E). To assess claudin18.2 expression levels, the paraffin sections were deparaffinized in xylene and rehydrated through a series of graded alcohols. Following antigen retrieval using a citrate acid solution, endogenous peroxidase activity was blocked with a 3% hydrogen peroxide (H₂O₂) solution. After blocking with 5% BSA, the slides were incubated with an anti-claudin18.2 primary antibody (rabbit, 1:500, Abcam) and then with a biotinylated secondary antibody (goat, 1:500, Yeasen). The slides were then treated with a 3,3′-diaminobenzidine chromogenic solution for 10 min and counterstained with H&E. The stained sections were examined under an optical microscope (Carl Zeiss), where the total number of PC cells and the number of claudin18.2^+^ PC cells were counted. The percentage of claudin18.2^+^ cells was calculated using the formula claudin18.2^+^ cell rate = (number of claudin18.2^+^ PC cells/total number of PC cells) × 100%.

### Generation of claudin18.2-targeted CAR-T cells

Autologous claudin18.2-targeted CAR-T cells were produced in a cyclic GMP-compliant clinical manufacturing facility, following the procedures previously described.^27^ Briefly, the process involved several key steps: collection of PB, enrichment of PB mononuclear cells, isolation and activation of T cells, transduction of T cells with a lentiviral vector encoding the claudin18.2 CAR, expansion of the CAR-T cells, and, ultimately, harvesting the CAR-T cells. The CAR construct consists of a humanized anti-claudin18.2 scFv, a CD8α hinge region, a 4-1BB co-stimulatory domain, and a CD3ζ signaling domain. Additionally, a nonfunctional tEGFR was included at the end of the CAR structure using a T2A linker, serving as both a marker for transduction and a safety switch. The CAR-T cells were further expanded in Lonza's X-VIVO medium supplemented with 10 ng/mL IL-7, 5 ng/mL IL-15, and 30 ng/mL IL-21 until the cell quantity met the required dose.

### Flow cytometry

To monitor the expansion of claudin18.2-targeted CAR-T cells, PB samples were collected at various time points. Following the lysis of red blood cells, the remaining cells were stained with a panel of anti-human antibodies, including EGFR-APC (BioLegend, 52906), CD3-APC/Cyanine7 (BioLegend, 300426), CD4-PerCP/Cyanine5.5 (BioLegend, 300530), CD45RA-FITC (BioLegend, 304106), CD197-PE (BioLegend, 353204), and CD8-PE/Cyanine7 (BioLegend, 344712). The claudin18.2-targeted CAR-T cells were then detected by flow cytometry. Since the claudin18.2-targeted CAR includes a tEGFR, these CAR-T cells could be directly identified using CD3-APC/Cyanine7 and tEGFR-APC antibodies. Additionally, the proportions of CD4^+^ CAR-T cells, CD8^+^ CAR-T cells, and central memory CD4^+^ T cells were assessed.

### scRNA library preparation and sequencing

PB samples (10 mL) were collected from patient 3 on days 14, 21, 31, and 54 post-infusion. White blood cells were isolated by red blood cell lysis, and T cells were subsequently enriched from both the infused product and these white blood cell samples using the MojoSort Human CD3 Selection Kit (BioLegend, 480134), following the manufacturer’s guidelines. The enriched cells were resuspended in PBS to create single-cell suspensions (1 × 10^5^ cells/mL), which were then loaded into microfluidic devices. scTCR-seq/scBCR (B cell receptor)-seq libraries were subsequently constructed using the GEXSCOPE Single Cell Immuno-TCR/BCR Kit (Singleron Biotechnologies). Briefly, magnetic beads with molecular labels captured the poly(A) tail and TCR regions on the mRNA, labeling both the cells and mRNA following cell lysis. The magnetic beads in the chip were then collected, and the captured mRNA was reverse transcribed into complementary DNA (cDNA) and amplified. Sequencing libraries, suitable for the Illumina platform, were constructed after partial cDNA fragmentation and splicing. The remaining cDNA was enriched for immune receptor (TCR) sequences, and the enriched products were amplified by PCR to construct a sequencing library compatible with the Illumina platform. Finally, each library was sequenced on the Illumina HiSeq X with 150-bp paired-end reads.

### scRNA-seq data processing

Sample demultiplexing, barcode processing, alignment to the human genome (GRCh38), and raw gene expression quantification were conducted using the Cell Ranger pipeline (version 6.0.1). The resulting raw single-cell expression matrices were further analyzed in R (version 4.3.2) utilizing the Seurat package (version 5.0.1). Quality control filters were applied, excluding cells with fewer than 200 genes, the top 2% of gene counts, and the top 2% of unique molecular identifier counts. Additionally, cells with over 25% mitochondrial content were removed. Doublets were identified using the DoubletFinder package with default settings and subsequently removed. Gene expression normalization was performed using the SCTransform function in Seurat. To address batch effects, the data were integrated using the SelectIntegrationFeatures, PrepSCTIntegration, FindIntegrationAnchors, and IntegrateData functions. Nonlinear dimensionality reduction was then performed using the RunTSNE function, and cluster analysis was conducted using the FindNeighbors and FindClusters functions, with clusters identified at a resolution of 0.1. CAR-T cells were distinguished by the expression of the lentivirus-inserted gene, marked as pCDHEXO.

TCR clonotype assignment was carried out using the Cell Ranger vdj pipeline (version 4.0.0) with GRCh38 as the reference. In this process, a TCR diversity metric, which includes clonotype frequency and barcode information, was obtained. Only cells with 1 productive TCR β chain (TRB) were retained for further analysis. Unique TRB(s) were defined as clonotypes, and clonotypes present in at least 2 cells were considered clonal, with the number of cells harboring such pairs indicating the degree of clonality.

To identify DEGs, the Seurat FindMarkers function was used, employing the Wilcoxon rank-sum test with default parameters. DEGs were defined as genes expressed in more than 20% of cells in 1 cluster compared to another, with an average log2 fold change greater than 2 and an adjusted *p* value below 0.05. Enrichment analysis of DEGs was performed using the clusterProfiler package, considering pathways with an adjusted *p* value of less than 0.05 as significantly enriched. Gene Ontology gene sets, including molecular function, biological process, and cellular component categories, were used as references.

## Data and code availability

The raw data supporting the conclusions of this article will be made available by the authors without undue reservation.

## Acknowledgments

We thank all the staff for the clinical support and technical support from the Department of Hematology and Oncology, Shenzhen University General Hospital, and Shenzhen University-Haoshi Cell Therapy Institute. This work was supported by Chinese National Maior Project for New Drug Innovation (2019ZX09201002003). National Natural Science Foundation of China (82030076, 81970151, 81670162, 82470229,82101928), Shenzhen Clinical Research Center for hematologic disease (LCYSSQ20220823091401002), Sanming Project of Medicine in Shenzhen (SZSM202111004), Shenzhen Key Laboratory Foundation (ZDSYS20200811143757022), Shenzhen Science and Technology Foundation (JCY120190808163601776,JCY120200109113810154), Shenzhen Key Laboratory Fund (ZDSYS20200811143757022), Medicine Plus Program of Shenzhen University (000003011601).

## Author contributions

L.Y., Y.L., and G.Z. conceptualized the manuscript. G.Z., X.Z., R.Z., Y.L., and C.Y. analyzed the data and wrote the manuscript. G.Z., C.W., Z.G., D.L., K.H., Y.G., B.Z., X.L., X.S., L.C., and L.Y. took care of the patients and collected the data. G.Z., R.Z., and X.Z. revised the manuscript. All authors read and approved the manuscript.

## Declaration of interests

The authors declare no competing interests.

## Supplemental information

Document S1. Figures S1–S7

Document S2. Article plus supplemental information
