# Supplementary material for: The high efficacy of claudin18.2-targeted CAR-T cell therapy in advanced pancreatic cancer with an antibody-dependent safety strategy
Source: Mol Ther. 2025 Jan 10;33(6):2778–88. doi: 10.1016/j.ymthe.2025.01.012 (PMC12172178; doi:10.1016/j.ymthe.2025.01.012)
Supplement: Document S1. Figures S1–S7 [file mmc1.pdf]

YMTHE, Volume *33*

Supplemental Information

The high efficacy of claudin18.2-targeted CAR-T cell therapy in advanced pancreatic cancer with an antibody-dependent safety strategy

Guocheng Zhong, Xiaomin Zhang, Ruocong Zhao, Zheng Guo, Chenguang Wang, Chuan Yu, Dongzhe Liu, Ke Hu, Yujie Gao, Bochen Zhao, Xianhao Liu, Xuanren Shi, Lei Chen, Yisheng Li, and Li Yu

Supplemental Figures

Figure S1

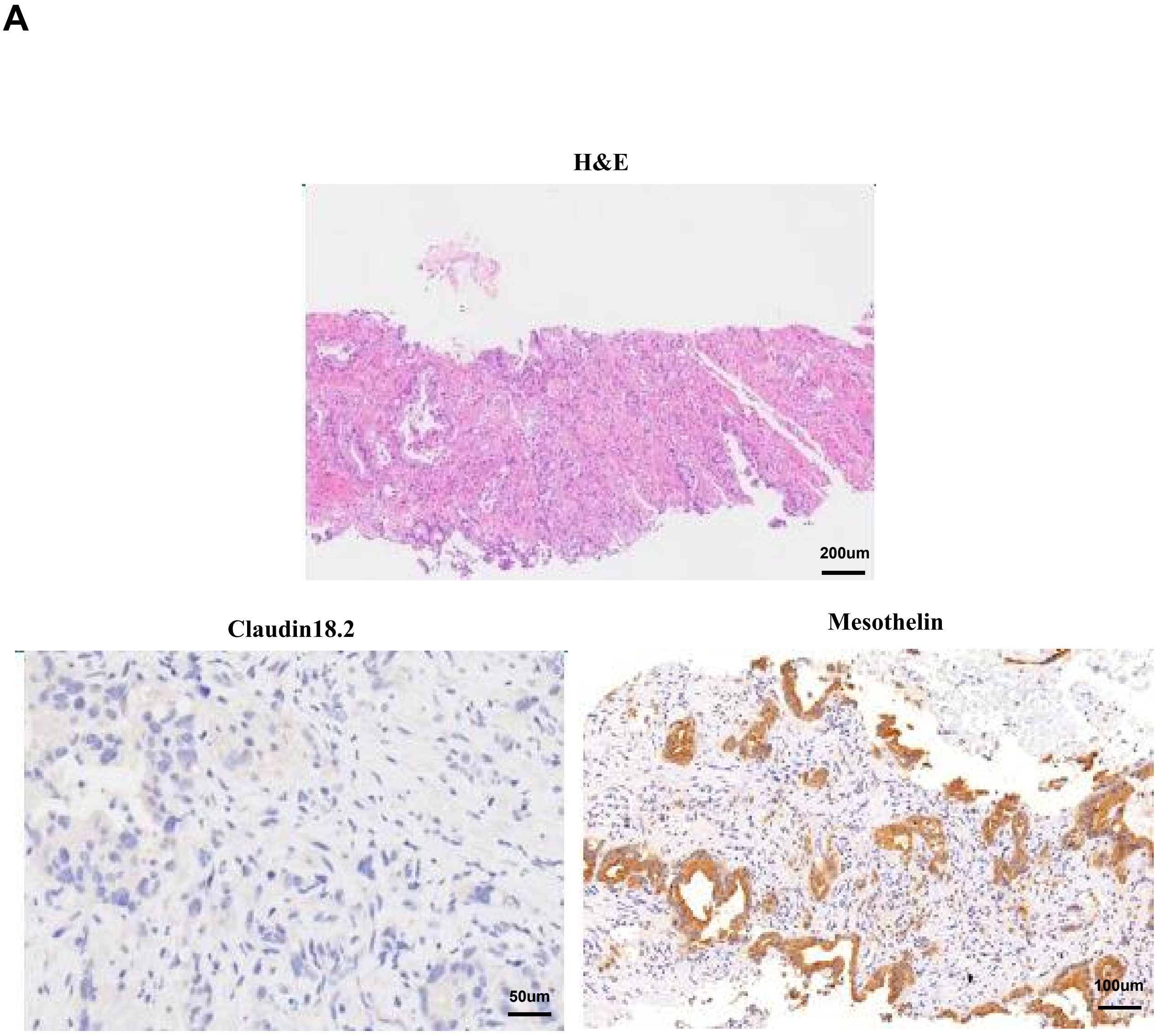

Figure S1. Antigen expression of relapsed tumor tissue in patient1

A) HE staining and Immunohistochemistry analysis of claudin18.2 and Mesothelin expression in relapsed tumor tissue sections from patient1.

Figure S2

A

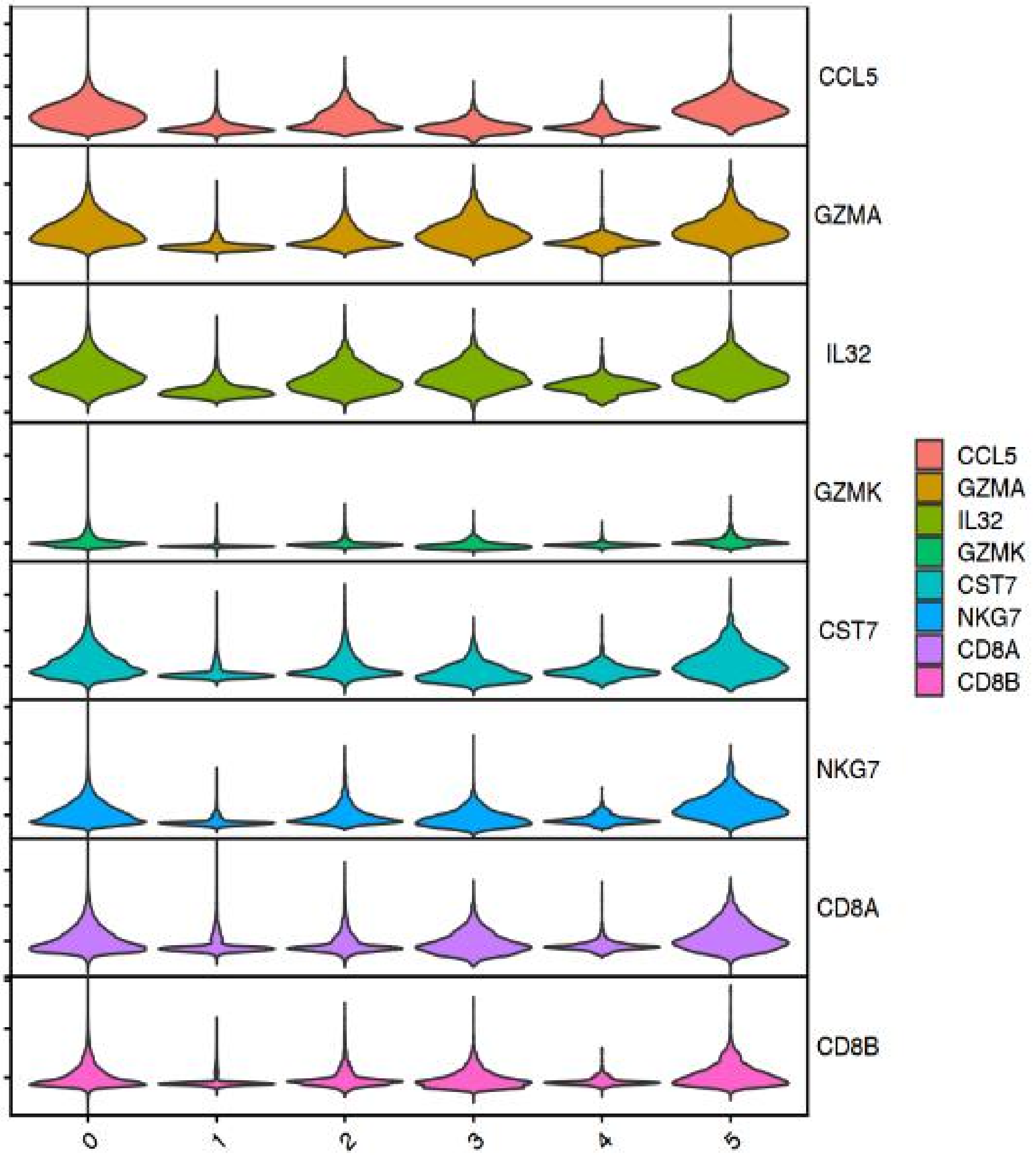
CCL5
GZMA
IL32
GZMK
CST7
NKG7
CD8A
CD8B
0
1
2
3
4
5
CCL5
GZMA
IL32
GZMK
CST7
NKG7
CD8A
CD8B

Figure S2. Violin Plots of T cell functional related Gene that highly expressed in clusters 0,2,3,5

A) Violin plots depicting the highly expressed key marker genes across CAR-T cell cluster 0,2,3,5. Each plot represents the distribution of gene expression within specific clusters identified from the scRNA-seq data.

Figure S3

A

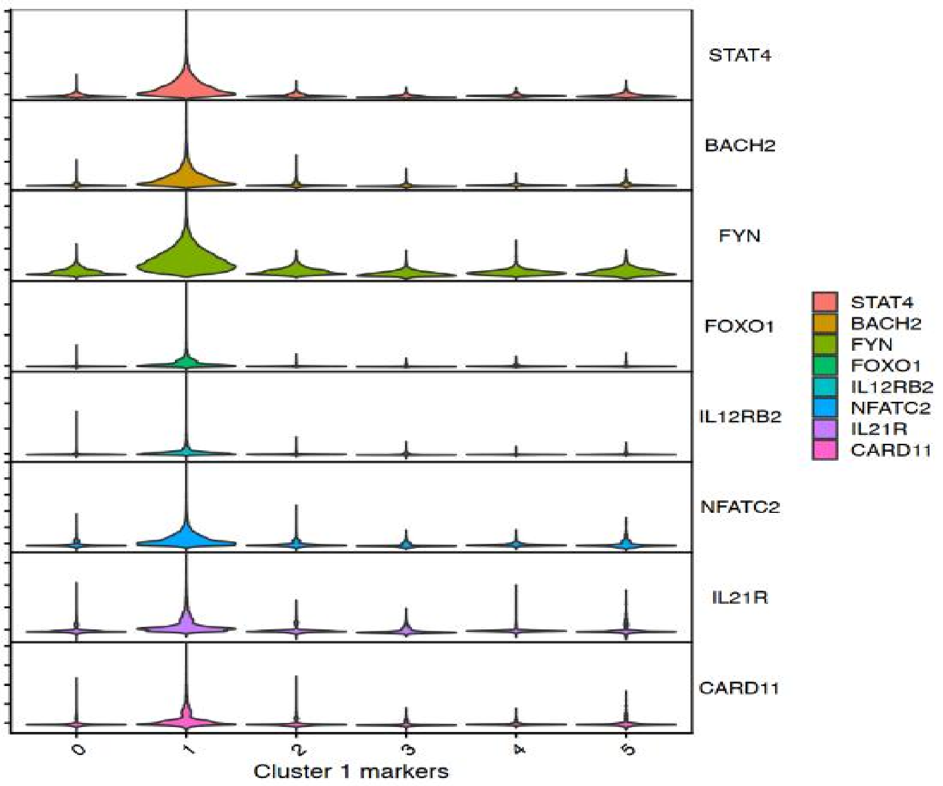
STAT4
BACH2
FYN
FOXO1
IL12RB2
NFATC2
IL21R
CARD11
0
1
2
3
4
5
Cluster 1 markers

B

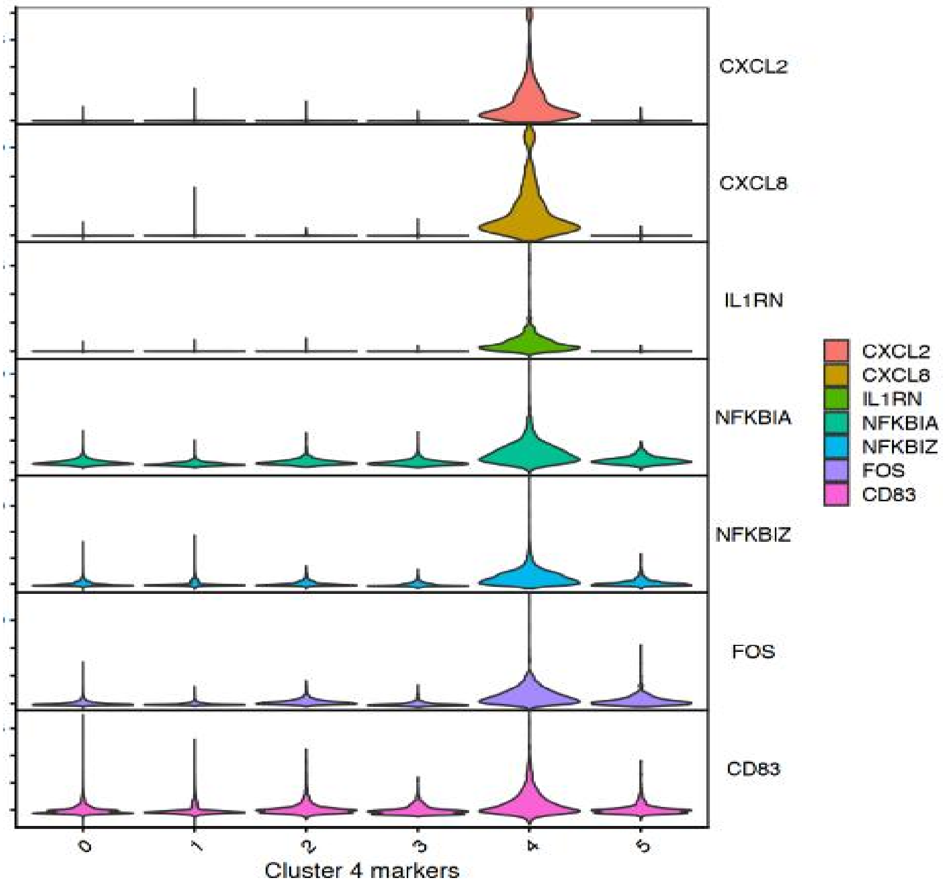
CXCL2
CXCL8
IL1RN
NFKBIA
NFKBIZ
FOS
CD83
0
1
2
3
4
5
Cluster 4 markers

Figure S3 Violin Plots of T cell functional related Gene that highly expressed in clusters 1 and 4

A) Violin plots depicting the highly expressed key marker genes of CAR-T cell cluster1. B) Violin plots depicting the highly expressed key marker genes of CAR-T cell cluster4.Each plot represents the distribution of gene expression within specific clusters identified from the scRNA-seq data.

.

Figure S4

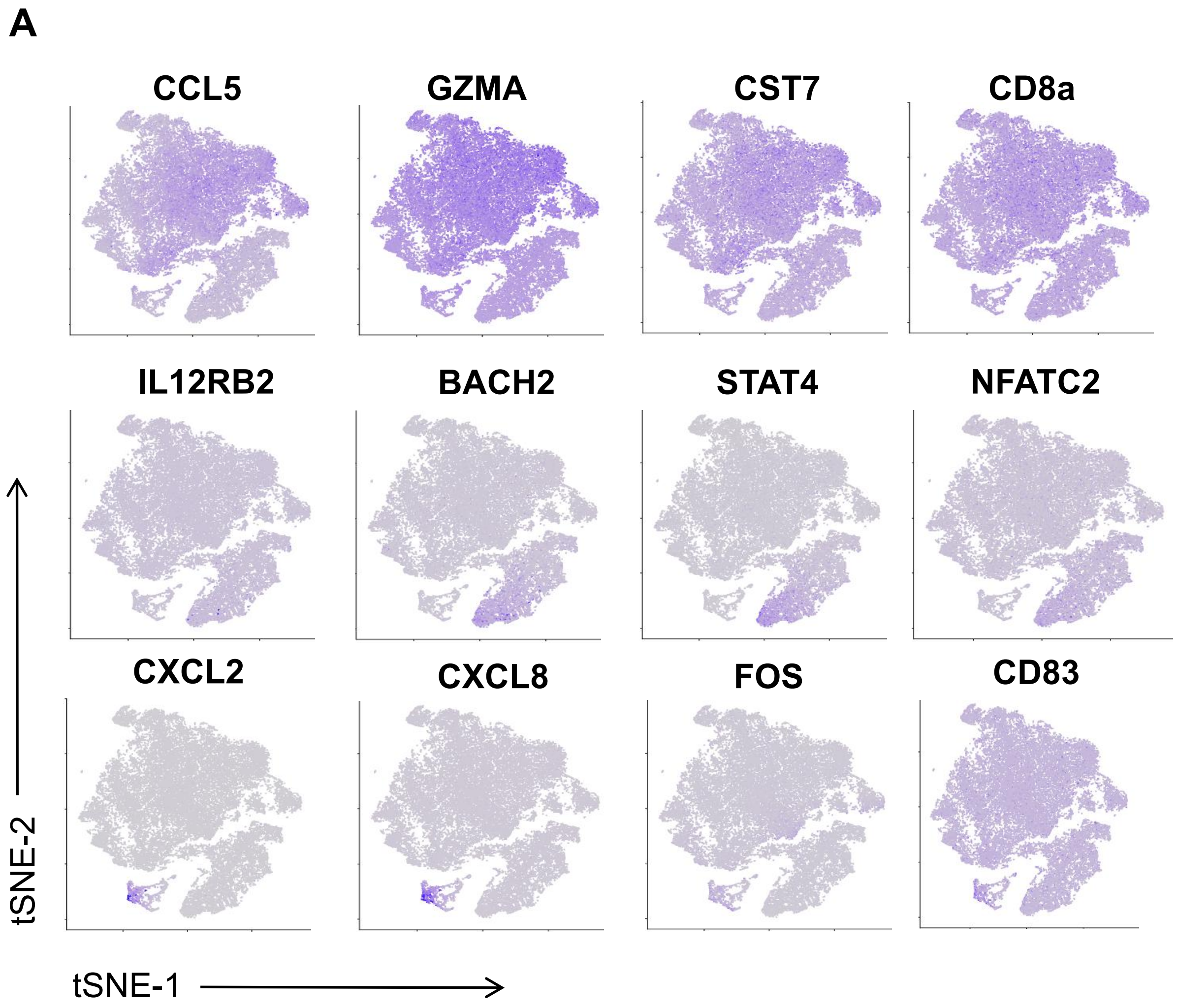
A
CCL5
GZMA
CST7
CD8a
IL12RB2
BACH2
STAT4
NFATC2
CXCL2
CXCL8
FOS
CD83
tSNE-2
tSNE-1

Figure S4 t-SNE analysis of single cell RNAseq data from CAR-T population

A) t-SNE plots showing the spatial distribution and expression levels of selected marker genes (CCL5, GZMA, CST7, CD8a, IL12RB2, BACH2, STAT4, NFATC2, CXCL2, CXCL8, FOS, CD83) across the CAR-T cell populations. The intensity of the color indicates the level of gene expression, with darker shades representing higher expression.

Figure S5

A

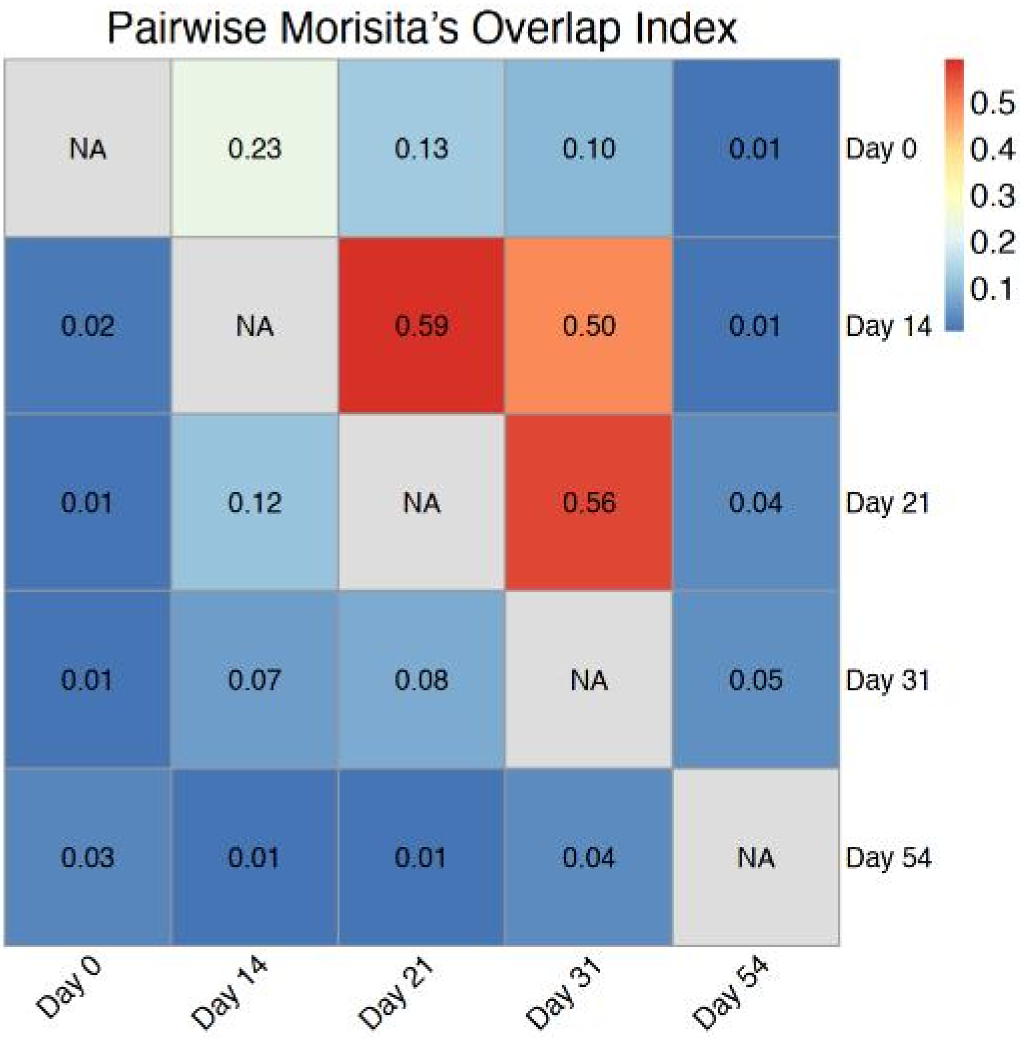

Pairwise Morisita's Overlap Index
NA
0.23
0.13
0.10
0.01
Day 0
0.02
NA
0.59
0.50
0.01
Day 14
0.01
0.12
NA
0.56
0.04
Day 21
0.01
0.07
0.08
NA
0.05
Day 31
0.03
0.01
0.01
0.04
NA
Day 54
Day 0
Day 14
Day 21
Day 31
Day 54
0.5
0.4
0.3
0.2
0.1

Figure S5 Analysis of TCR repertoire diversity

A) pairwise Morisita's Overlap index analysis for the baseline variability in TCR repertoires among different CAR-T cell samples.

Figure S6

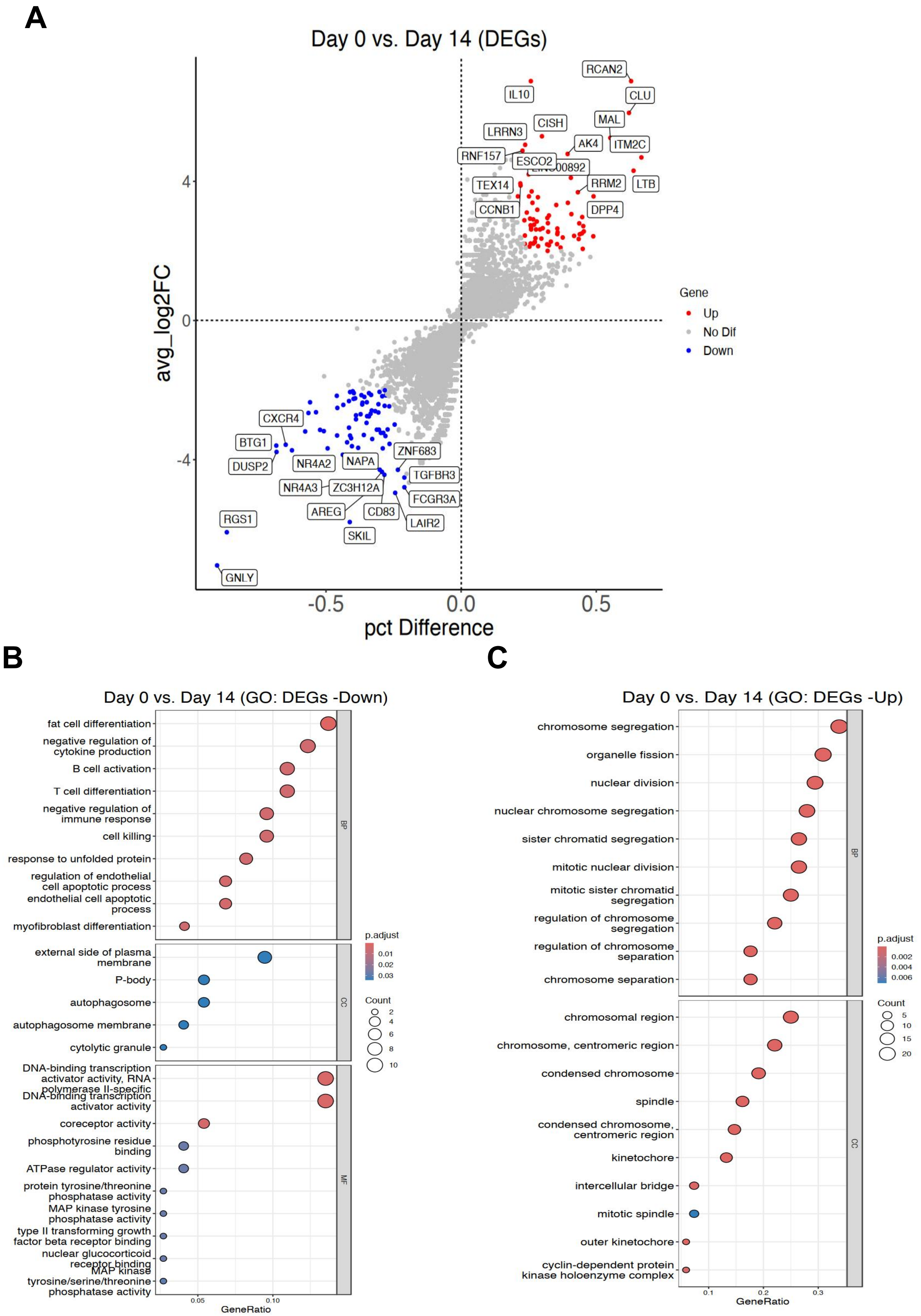
A
Day 0 vs. Day 14 (DEGs)
avg_log2FC
pct Difference
Gene
Up
No Dif
Down
RCAN2
CLU
IL10
MAL
CISH
LRRN3
AK4
ITM2C
RNF157
ESCO2
LTB
RRM2
TEX14
DPP4
CCNB1
CXCR4
BTG1
DUSP2
NR4A2
NAPA
ZNF683
TGFBR3
NR4A3
ZC3H12A
FCGR3A
AREG
CD83
LAIR2
RGS1
SKIL
GNLY
B
Day 0 vs. Day 14 (GO: DEGs -Down)
fat cell differentiation
negative regulation of cytokine production
B cell activation
T cell differentiation
negative regulation of immune response
cell killing
response to unfolded protein
regulation of endothelial cell apoptotic process
endothelial cell apoptotic process
myofibroblast differentiation
external side of plasma membrane
P-body
autophagosome
autophagosome membrane
cytolytic granule
DNA-binding transcription activator activity, RNA polymerase II-specific
DNA-binding transcription activator activity
coreceptor activity
phosphotyrosine residue binding
ATPase regulator activity
protein tyrosine/threonine phosphatase activity
MAP kinase tyrosine phosphatase activity
type II transforming growth factor beta receptor binding
nuclear glucocorticoid receptor binding
MAP kinase tyrosine/serine/threonine phosphatase activity
GeneRatio
p.adjust
Count
C
Day 0 vs. Day 14 (GO: DEGs -Up)
chromosome segregation
organelle fission
nuclear division
nuclear chromosome segregation
sister chromatid segregation
mitotic nuclear division
mitotic sister chromatid segregation
regulation of chromosome segregation
regulation of chromosome separation
chromosome separation
chromosomal region
chromosome, centromeric region
condensed chromosome
spindle
condensed chromosome, centromeric region
kinetochore
intercellular bridge
mitotic spindle
outer kinetochore
cyclin-dependent protein kinase holoenzyme complex
GeneRatio
p.adjust
Count

Figure S6. Clonal evolution and differential gene expression for CAR-T cell at Day0 and Day14

A) Comparion of Day 0 versus Day 14 CAR-T cells for their differentially expressed genes (DEGs) within the pre-existing TCR clones B) Down-regulated and C) up-regulated pathways of Gene Ontology (GO) enrichment analyses for CAR-T cells at Day 0 (D0) and Day 14 (D14) post-expansion.

Figure S7

A

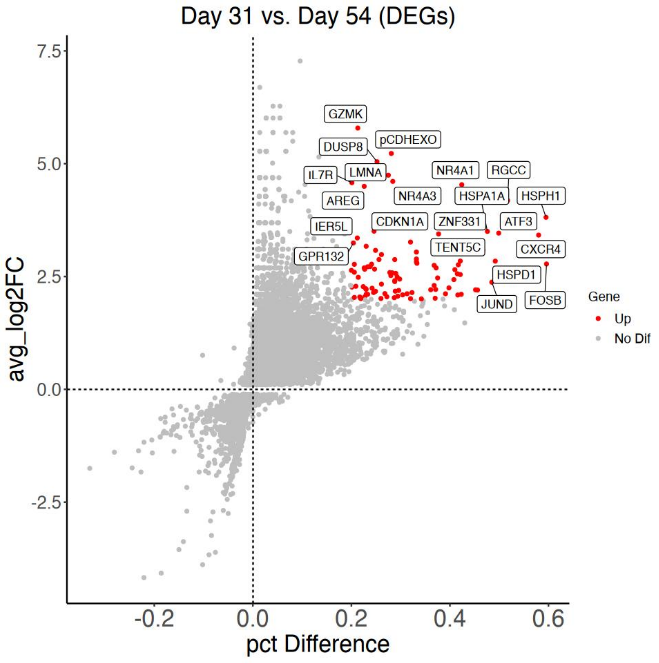

Day 31 vs. Day 54 (DEGs)
avg_log2FC
pct Difference
Gene
Up
No Dif
GZMK
pCDHEXO
DUSP8
IL7R
LMNA
NR4A1
RGCC
AREG
NR4A3
HSPA1A
HSPH1
IER5L
CDKN1A
ZNF331
ATF3
GPR132
TENT5C
CXCR4
HSPD1
JUND
FOSB

B

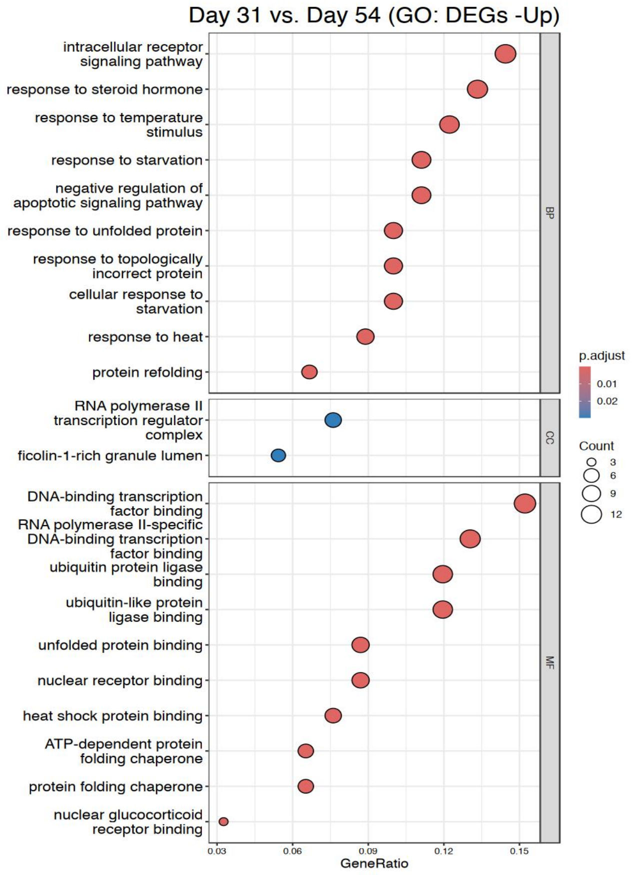

Day 31 vs. Day 54 (GO: DEGs -Up)
intracellular receptor signaling pathway
response to steroid hormone
response to temperature stimulus
response to starvation
negative regulation of apoptotic signaling pathway
response to unfolded protein
response to topologically incorrect protein
cellular response to starvation
response to heat
protein refolding
RNA polymerase II transcription regulator complex
ficolin-1-rich granule lumen
DNA-binding transcription factor binding
RNA polymerase II-specific DNA-binding transcription factor binding
ubiquitin protein ligase binding
ubiquitin-like protein ligase binding
unfolded protein binding
nuclear receptor binding
heat shock protein binding
ATP-dependent protein folding chaperone
protein folding chaperone
nuclear glucocorticoid receptor binding
BP
CC
MF
GeneRatio
p.adjust
Count

Figure S7. Clonal evolution and differential gene expression for CAR-T cell at Day 31 and Day 54

A) Comparion of Day 31 versus Day 54 CAR-T cells for their differentially expressed genes (DEGs) within the pre-existing TCR clones B) Down-regulated pathways of Gene Ontology (GO) enrichment analyses for CAR-T cells at Day 0 (D0) and Day 14 (D14) post-expansion.
